# Alkane hydroxylase genes in psychrophile genomes and the potential for cold active catalysis

**DOI:** 10.1186/1471-2164-15-1120

**Published:** 2014-12-16

**Authors:** Jeff S Bowman, Jody W Deming

**Affiliations:** School of Oceanography and Astrobiology Program, University of Washington, Box 357940, Seattle, WA 98105-7940 USA; Blue Marble Space Institute of Science, Seattle, USA

## Abstract

**Background:**

Psychrophiles are presumed to play a large role in the catabolism of alkanes and other components of crude oil in natural low temperature environments. In this study we analyzed the functional diversity of genes for alkane hydroxylases, the enzymes responsible for converting alkanes to more labile alcohols, as found in the genomes of nineteen psychrophiles for which alkane degradation has not been reported. To identify possible mechanisms of low temperature optimization we compared putative alkane hydroxylases from these psychrophiles with homologues from nineteen taxonomically related mesophilic strains.

**Results:**

Seven of the analyzed psychrophile genomes contained a total of 27 candidate alkane hydroxylase genes, only two of which are currently annotated as alkane hydroxylase. These candidates were mostly related to the AlkB and cytochrome p450 alkane hydroxylases, but several homologues of the LadA and AlmA enzymes, significant for their ability to degrade long-chain alkanes, were also detected. These putative alkane hydroxylases showed significant differences in primary structure from their mesophile homologues, with preferences for specific amino acids and increased flexibility on loops, bends, and α-helices.

**Conclusion:**

A focused analysis on psychrophile genomes led to discovery of numerous candidate alkane hydroxylase genes not currently annotated as alkane hydroxylase. Gene products show signs of optimization to low temperature, including regions of increased flexibility and amino acid preferences typical of psychrophilic proteins. These findings are consistent with observations of microbial degradation of crude oil in cold environments and identify proteins that can be targeted in rate studies and in the design of molecular tools for low temperature bioremediation.

**Electronic supplementary material:**

The online version of this article (doi:10.1186/1471-2164-15-1120) contains supplementary material, which is available to authorized users.

## Background

In the natural environment crude oil, a complex mixture of light and heavy hydrocarbons and inorganic compounds, is degraded by members of the Bacteria and Archaea, as well as by certain plants and fungi. Significant work has been done to identify the taxonomic groups and pathways involved in the bioremediation of crude oil, motivated in part by the need to improve predictions of *in situ* degradation rates of oil and of targeted hydrocarbon compounds. The 2010 Macondo Well blowout in the Gulf of Mexico highlighted some of the gaps in our understanding of how crude oil is degraded *in situ* in the pelagic marine environment, as both the hydrocarbons emerging from the damaged well and the *in situ* marine microbial community reacted in ways that were unpredicted [1–3]. In that case, components of the crude oil advecting in a deep plume below 1000 m in the Gulf were consumed *in situ* by the marine microbial community, reducing ecological disturbance at the sea surface.

The extent of the microbial crude oil catabolism at the relatively cold temperature (approximately 5°C) of the deep plume was considered surprising [2]. It has long been recognized, however, that bacteria can respond quickly to crude oil in near-freezing seawater [4]. Even sea ice microbial communities, living at temperatures below the freezing point of seawater, can respond to inputs of diesel fuel and crude oil [5–7]. Low temperature crude oil degradation has also been observed in polar and alpine soil [8–10], and by several Bacterial strains in culture [11–13]. Despite these and other advances in understanding the potential for low temperature bioremediation, the presence of crude oil degradation genes in the available psychrophile genomes has not been investigated, though recent work has suggested that these genes might be broadly distributed across the Bacteria and Archaea [14]. By identifying such genes and evaluating differences between gene products and homologues from mesophiles, we hoped to identify structural differences that may enable crude oil catabolism at low temperatures. In addition to improving our ability to predict *in situ* bioremediation in cold environments, this knowledge paves the way for the rational design or modification of enzymes for improved function at *in situ* temperature in polar and sub-polar environments. These considerations are important for small scale, reduced energy, environmental clean-up strategies involving bioreactors and other technologies. Rational protein manipulation has already resulted in enzymes of potential value for environmental cleanup and industrial processes [15, 16]; however, this work has been limited to possible terrestrial, not marine, applications at standard conditions for temperature and pressure.

By mass a considerable fraction of crude oil is n-alkanes (alkanes): straight chain, saturated hydrocarbons with no cyclic functional groups. The shortest and most volatile alkanes are the natural gas components methane, ethane, butane, and propane, all of which are important substrates for a variety of Bacteria and Archaea. Even approaching the freezing point of water these small alkanes remain in the gas phase and are therefore highly bioavailable. Bioavailability decreases with the increasing number of carbons in an alkane molecule, reaching a minimum with large, extremely hydrophobic waxes [4]. At mesophilic growth temperatures alkanes larger than C_16_ are solid, necessitating the use of emulsifiers to improve bioavailability [17]. To degrade alkanes of different lengths Bacteria and Archaea have evolved a diverse array of enzymes, collectively termed alkane hydroxylases. All alkane hydroxylases function by oxidizing the terminal or subterminal carbon, converting the alkane into an alcohol [18]. This conversion “activates” the alkane for processing by downstream enzymes, starting with alcohol dehydrogenase.

The diversity of alkane hydroxylases, described in recent reviews [17–20], is briefly summarized here. Operating on the lowest molecular weight alkanes (approximately C_1_-C_4_) are the soluble methane monooxygenase (SMMO), particulate methane monooxygenase (PMMO), and propane/butane monooxygenase (P/BMO) enzymes. Acting on mid-weight alkanes (roughly C_5_-C_16_) are a group of alkane hydroxylases belonging to the cytochrome p450 family of enzymes and the membrane-bound non-heme AlkB enzymes. Less is known about the degradation of long chain alkanes, but two enzymes, AlmA and LadA, have been identified that utilize alkanes large than C_20_ [21, 22].

To explore the diversity of alkane hydroxylases in the genomes of psychrophilic Bacteria we conducted a *de novo* annotation of nineteen psychrophile genomes, searching for homologues of known alkane hydroxylase genes. To evaluate what properties of these proteins might enable catalytic function at low temperature we compared protein parameters between putative alkane hydroxylases from psychrophiles and mesophiles averaged across the whole protein, within secondary structure elements, and, for protein flexibility, within specific residues along the length of the protein.

## Methods

### Identifying alkane hydroxylases

Proteins representative of alkane hydroxylases were identified in the Universal Protein Resource (Uniprot) database [23] by protein name search for ‘alkane hydroxylase’ , ‘methane monooxygenase’ , ‘propane monoxygenase’ , ‘butane monooxygenase’ , ‘LadA’ , and ‘AlmA’. Proteins belonging to uncultured organisms or identified as fragments were excluded from further analysis, while duplicated names or sequences were reduced to a single copy. An exception was made to allow fragments for AlmA, as all AlmA proteins in the database were described as fragments yet were of similar length. Conserved domains were identified in the representative alkane hydroxylases by hmmscan in HMMER v3.0 [24] against the PFAM-A database of protein families [25] with an e-value cutoff of 10^−5^. Hmmscan uses profile-hidden Markov models, a representation of amino acid probability by position, to match query sequences against a database.

Nineteen psychrophile strains (maximum growth temperature < 20°C) and nineteen closely related mesophile strains were selected and their genomes downloaded from Genbank (Additional file 1: Figure S1). Psychrophiles were identified according to temperature annotations in the GOLD [26] and HIMA [27] databases, and to reviews by Casanueva et al. [28] and Siddiqui et al. [29]. Care was taken to include all plasmids and chromosomes with the genome of each strain. Open reading frames (ORFs), defined as any region longer than 150 bp without a stop codon, were translated and searched for conserved protein domains against the PFAM-A database [25] using hmmscan in HMMER v3.0 [24] and an E-value cutoff of 10^−5^. Coding sequences (CDS, ORFs containing a pfam domain) with a hit to a pfam present in alkane hydroxylases were extracted for further analysis.

Complete records for diagnostic pfams were downloaded as fasta files from the Protein Family website (PFAM; http://pfam.xfam.org/). For PFAM datasets larger than 5,000 sequences, 5,000 sequences were randomly selected for analysis. For each pfam, the PFAM dataset was combined with the proteins of that family from the Uniprot, psychrophile, and mesophile datasets. These combined protein sets were aligned using three iterative alignments in Clustal Omega v1.2 [30], a program that allows for high-quality alignments of large numbers of protein sequences. The alignments were then filtered using an in-house script (filter_seqs_selective.py) which trims the alignment to the last start and earliest end position of the proteins from the Uniprot dataset. Proteins from the psychrophile, mesophile, or PFAM datasets that did not meet a minimum length guideline after filtering were eliminated from further analysis. After filtering, the sequences were aligned one more time, and a distance matrix of each pfam was created using the --full and --use-kimura flags in Clustal Omega v1.2.

To describe sequence similarity within pfams we used nonmetric multidimensional scaling (NMDS) of Kimura-corrected genetic distance [31] in the R package Vegan [32]. This method was selected over phylogenetic trees based on the ease with which points in a region of interest on a 2D NMDS plot can be selected programmatically, compared to selecting branches on a phylogenetic tree. Although NMDS plots have been used to describe protein homology previously [33], this method is not in wide use. To validate the NDMS approach to describing sequence similarity we compared the Euclidean distance between NMDS points in the first and second dimension, maximum likelihood distance from a phylogenetic tree based on the same alignment, and bit scores from a reciprocal blastp search. We used the combined protein dataset for the FA_desaturase pfam for this analysis and generated a tree of the filtered alignment using FastTree OpenMP v2 [34] with the JTT+CAT model. Summed branch lengths between all branch tips were extracted from the tree with an in-house script (dist_from_tree.py) using the Phylo package in Biopython [35]. To describe the relationship between phylogenetic tree distance, bit score, and NMDS distance, linear models were fit to a randomly selected subset of the data (n = 10,000) in log-linear space for NMDS and phylogenetic distance and log-log space for NMDS and bit score distance. Goodness of fit was further evaluated by exploring the distribution of the residuals.

For NMDS analysis we determined the ideal number of dimensions to be three for fewer than 3,000 sequences, four for between 3,000 and 6,000 sequences, and five for more than 6,000 sequences. Sequences that placed far from the majority of points in a 2D plot of the NMDS analysis, and thus prevented the identification of distinct clusters for the majority of points, were culled from the original alignment and a new distance matrix was constructed before re-running the NMDS analysis. Clusters on the final 2D NMDS plots that contained proteins from the Uniprot, psychrophile, and mesophile datasets were selected for further analysis.

### Analysis of protein parameters

The flexibility, grand average of hydropathy (GRAVY), isoelectric point, and aromaticity parameters of proteins were calculated with the ProtParam module in BioPython [35]. Aliphatic index was calculated using the method of Ikai [36]. To determine the parameters by secondary structure, the α-helix, β-strand, and coil region for each protein was determined by the stand-alone version of psipred [37] and the runpsipredplus script. The best database for secondary structure prediction was evaluated by comparing predictions using the NCBI nr database, uniref90, and Pfam-A for one candidate alkane hydroxylase against predictions obtained from an intensive 3-D structural prediction model using Phyre2 [38]. Both databases achieved a prediction accuracy of 71.8%, just below the prediction of psipred as implemented by the Phyre2 server (72.7%). We used Pfam-A for further predictions due to the smaller size of that database. Protein parameters were recalculated using a 9 residue window (selected for consistency with the window used in flexibility calculation), and the per-position parameter was taken as the mean of the window centered on that position. Per-position values for each parameter were then extracted for comparison according to secondary structure. Differences in parameters between psychrophile and mesophile proteins within clusters and secondary structure elements were evaluated using the Wilcox test. Differences in parameters between psychrophile and mesophile proteins within taxonomic pairs were evaluated with a pairwise comparison. Because multiple parameters were investigated, all p-values derived from the Wilcox Test were corrected for multiple comparisons using the Holm-Bonferroni method.

To evaluate differences in flexibility, widely considered important for cold activity, between putative alkane hydroxylases from psychrophiles and mesophiles on a by-position basis, we aligned the flexibility parameters for all proteins in each cluster according to a multiple sequence alignment generated in Clustal Omega v2 [30] using in-house scripts (align_params.py, align_params.r). For each position in the alignment the mean flexibility and standard deviation were calculated for psychrophile and mesophile proteins. Positions in the alignment where the difference in means (psychrophile proteins – mesophile proteins) between the two groups exceeded the sum of the standard deviations were flagged as sites of significant deviation. To place these findings in the context of protein tertiary structure, 3D models were constructed of a representative psychrophile protein in each cluster using the intensive modeling option in Phyre2 [38]. Residues with significant differences in flexibility were color-highlighted in the models using Discovery Studio Visualizer (Accelyrs).

All in-house scripts can be obtained from https://github.com/bowmanjeffs/cold_ah.

## Results

**Figure 1:**
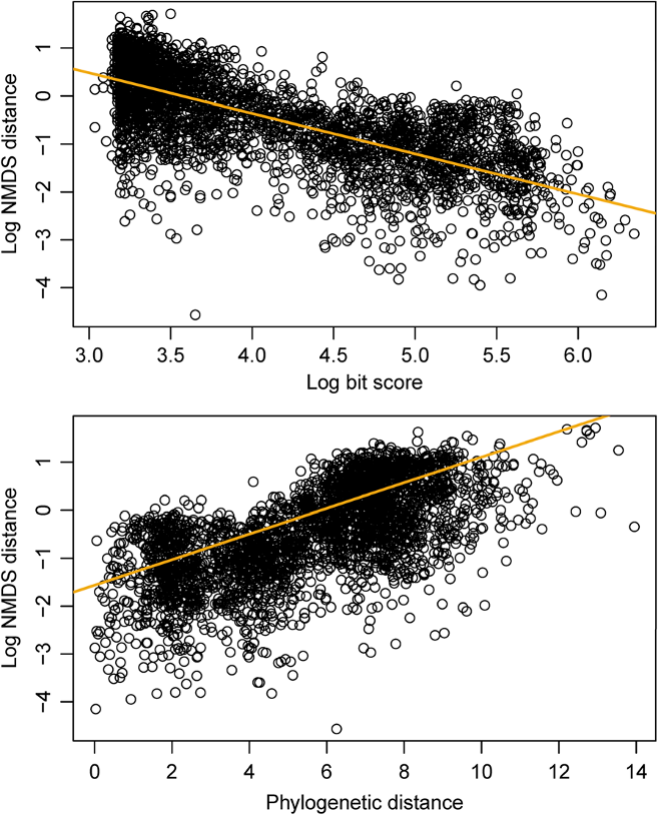
**Euclidean distance in 2D NMDS space as a function of bit score (top) and phylogenetic distance (bottom).** Euclidean distance was compared to bit score and phylogenetic distance to evaluate the fidelity of these parameters. Euclidean distance in the FA_desaturase pfam is strongly correlated with bit score (R^2^ = 0.4232 n = 2,439,512), obtained from reciprocal blast, and with phylogenetic distance (R^2^ = 0.5029, n = 2,439,512), as summed branch lengths from a maximum-likelihood tree. Orange lines are linear models fit to the complete data sets; only 10,000 randomly selected data points are plotted.

**Figure 2:**
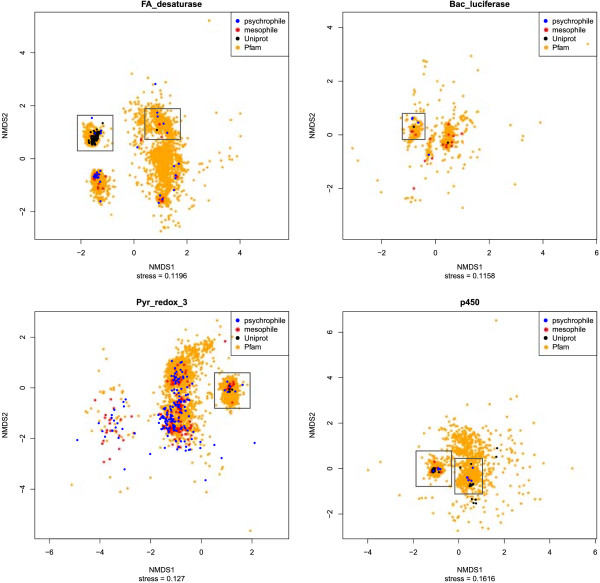
**NMDS plots of genetic distance within four protein families (pfams).** The distance between two points on the plot is proportional to their sequence similarity, thus neighboring points have similar functions. Clusters of points identified as candidate alkane hydroxylases, due to the presence of known alkane hydroxylases from Uniprot, are outlined with gray boxes.

The Uniprot searches collectively returned 939 alkane hydroxylase proteins after culling. These proteins belonged to 16 pfams of which seven were determined to have a regulatory or electron carrier binding function, or to be the result of an erroneous classification (MmoB_DmpM, ADH_zinc, LXG, Nol1_Nop2_Fmu, DUF900, NAD_binding_1, FAD_binding_6). Four of the remaining nine pfams were represented in the psychrophile genomes (Table 1). Among these four was FA_desaturase, used to show the correlations between Euclidean distance in 2D NMDS space and phylogenetic tree distance (R^2^ = 0.4232) and bit score (R^2^ = 0.5029) and thus the validity of using NMDS plots (Figure 1). NMDS plots of all four pfams contained clusters with both psychrophilic and Uniprot proteins, indicating close sequence similarity (Figure 2, Table 2). The Pyr_redox_3 and Bac_luciferase pfams each had only one cluster, corresponding to AlmA and LadA respectively. FA_desaturase had two clusters; cluster 0 corresponds to the AlkB group of membrane bound alkane hydroxylases, while cluster 1 is defined by only a single Uniprot protein annotated as alkane-1 monooxygenase. The p450 family also had two clusters; cluster 0 corresponds to the Bacterial p450 alkane hydroxylase, while cluster 1 corresponds to the Eukaryotic p450 alkane hydroxylase. A total of 26 putative alkane hydroxylases were identified in the psychrophile genomes and 41 in the mesophile genomes (Additional file 2: Table S1).

**Table 1:** **Occurrence of conserved protein family (pfam) domains linked to alkane hydroxylases (AH) in each dataset**

pfam domain	AH example	Psychrophile	Mesophile	Uniprot	AH candidate, psychrophile	AH candidate, mesophile
^1^AMO	^10^PMMO subunit A	0	0	48	0	0
^2^AmoC	PMMO subunit C	0	0	63	0	0
^3^Bac_luciferase	LadA	18	51	7	3	4
^4^FA_desaturase	AlkB	32	40	320	8	10
^5^MeMO_Hyd_G	^11^SMMO subunit G	0	0	17	0	0
^6^Monooxygenase_B	PMMO subunit B	0	0	46	0	0
^7^p450	p450	11	10	145	9	9
^8^Phenol_Hydrox	^12^PMO small subunit	0	0	164	0	0
^9^Pyr_redox_3	AlmA	176	196	35	6	18

Abbreviations for pfam are: ^1^ammonia monooxygenase, ^2^ammonia monooxygenase subunit C, ^3^Bacterial luciferase, ^4^fatty acid desaturase, ^5^methane monooxygenase hydrolase chain G, ^6^monooxygenase subunit B, ^7^cytochrome p450, ^8^phenol hydroxylase, and ^9^pyridine nucleotide-disulphide oxidoreductase; for AH example are: ^10^particulate methane monooxygenase, ^11^soluble methane monooxygenase, and ^12^propane monooxygenase.

**Table 2:** **Number of candidate alkane hydroxylases observed in each of the psychrophile and mesophile genomes examined**

Strain	Pair	Accession	LadA	AlmA	AlkB	Uncharacterized AlkB-like	Bacterial p450	Eukaryotic p450
**Psychrophiles**								
^1^ *Aeromonas salmonicida* A449	A	CP000644	0	0	0	0	0	0
^1^ *Aliivibrio salmonicida* LFI1238	B	FM178379	0	0	0	0	0	0
^2^ *Colwellia psychrerythraea* 34H	C	CP000083	0	0	0	0	0	0
^3^ *Desulfotalea psychrophila* LSv54	D	CR522870	0	0	0	0	0	0
^1^ *Flavobacterium psychrophilum* JIP02 86	E	AM398681	0	0	0	0	0	0
^**4**^ ***Glaciecola psychrophila*** **170**	**F**	**CP003837**	**0**	**5**	**0**	**1**	**1**	**0**
^5^ *Methanococcoides burtonii* DSM 6242	G	CP000300	0	0	0	0	0	0
^**4**^ ***Octadecabacter antarcticus*** **307**	**H**	**CP003740**	**1**	**0**	**2**	**2**	**1**	**1**
^**4**^ ***Octadecabacter arcticus*** **238**	**I**	**CP003742**	**1**	**0**	**2**	**1**	**1**	**1**
^**6**^ ***Photobacter profundum*** **SS9**	**J**	**CR354532**	**0**	**0**	**0**	**0**	**0**	**1**
^7^ *Pseudoalteromonas haloplanktis* TAC125	K	CR954246	0	0	0	0	0	0
^3^ *Psychrobacter arcticum* 273-4	L	CP000082	0	0	0	0	0	0
^**3**^ ***Psychrobacter cryohalolentis*** **K5**	**M**	**CP000323**	**0**	**1**	**0**	**0**	**0**	**0**
^**4**^ ***Psychroflexus torquis*** **ATCC700755**	**N**	**CP003879**	**0**	**0**	**0**	**0**	**0**	**1**
^4^ *Psychromonas* CNPT3	O	CP004404	0	0	0	0	0	0
^6^ *Psychromonas ingrahamii* 37	P	CP000510	0	0	0	0	0	0
^7^ *Shewanella halifaxensis* HAW EB4	Q	CP000931	0	0	0	0	0	0
^2^ *Shewanella sediminis* HAW-EB3	R	CP000821	0	0	0	0	0	0
^6^ *Shewanella violacea* DSS12	S	AP011177	0	0	0	0	0	0
^**8**^ ***Terroglobus saanensis*** **SP1PR4**	**T**	**CP002467**	**1**	**0**	**0**	**0**	**2**	**1**
**Mesophiles**								
*Aeromonas veronii* B565	A	CP002607	0	0	0	0	0	0
*Vibrio fischeri* ES114	B	CP000020	0	0	0	0	0	0
***Alteromonas macleodii*** **Deep ecotype**	**C**	**CP001103**	**0**	**1**	**0**	**0**	**0**	**0**
*Desulfocapsa sulfexigens* DSM 10523	D	CP003985	0	0	0	0	0	0
*Flavobacterium indicum* GPTSA100 9	E	HE774682	0	0	0	0	0	0
***Glaciecola agarilytica*** **4H37Ye5**	**F**	**CP002526**	**0**	**1**	**0**	**0**	**3**	**0**
*Methanosarcina mazei* Tuc01	G	CP004144	0	0	0	0	0	0
***Rhodobacter sphaeroides*** **ATCC 17025**	**H**	**CP000661**	**0**	**0**	**1**	**0**	**1**	**0**
***Ketogulonicigenium vulgare*** **Y25**	**I**	**CP002224**	**0**	**1**	**0**	**0**	**0**	**1**
*Vibrio vulnificus* YJ016	J	AP005352	0	0	0	0	0	0
***Pseudoalteromonas atlantica*** **T6c**	**K**	**CP000388**	**0**	**0**	**0**	**0**	**1**	**0**
***Acinetobacter baumannii*** **ACICU**	**L**	**CP000863**	**2**	**4**	**1**	**1**	**0**	**0**
***Acinetobacter baumannii*** **AYE**	**M**	**CU459141**	**2**	**6**	**1**	**1**	**0**	**0**
***Flavobacteriales bacterium*** **HTCC2170**	**N**	**CP002157**	**0**	**0**	**1**	**0**	**0**	**0**
***Marinobacter aquaeolei*** **VT8**	**O**	**CP000514**	**0**	**3**	**3**	**1**	**2**	**0**
***Alteromonas macleodii*** **English Channel ecotype**	**P**	**CP003844**	**0**	**1**	**0**	**0**	**0**	**0**
*Shewanella* MR-7	Q	CP000444	0	0	0	0	0	0
***Shewanella denitrificans*** **OS217**	**R**	**CP000302**	**0**	**1**	**0**	**0**	**0**	**0**
*Shewanella putrefacien*s 200	S	CP002457	0	0	0	0	0	0
***Terriglobus roseus*** **DSM18391**	**T**	**CP003379**	**0**	**0**	**0**	**0**	**0**	**1**

Letters in the pair column indicate the taxonomic match used for the pairwise analysis. Strains with putative alkane hydroxylase genes indicated by bold.

Isolation environments for psychrophile strains are: ^1^metazoan host, ^2^sediment, ^3^permafrost, ^4^sea ice, ^5^saline lake, ^6^deep sea, ^7^surface seawater, ^8^tundra soil.

The Pyr_redox_3, Bacterial_luciferase, FA_desaturase, and p450 families contained sufficient psychrophile and mesophile proteins to allow a comparison of protein parameters within these two families. Considering parameters averaged across the protein, no pfam had a statistically significant difference in any parameter between the psychrophile and mesophile populations. We use the term “trending” to describe possible relationships between parameters and temperature class when p << 0.05 by the Wilcox Test but differences did not meet the significance threshold after applying the Holm-Bonferroni method to correct for multiple comparisons [39]. Trending differences were observed in three of the four pfams. Flexibility and tryptophan content trended lower in psychrophile FA_desaturase, while GRAVY and lysine content trended higher. For p450, threonine content trended higher in psychrophiles. For Bac_luciferase, alanine, isoleucine, and lysine trended lower in psychrophiles, while cysteine, methionine, arginine, and tyrosine trended higher.

Comparing between taxon pairs (as given in Table 2) revealed more differences for parameters averaged across the whole protein (Table 3). For p450, four psychrophile-mesophile taxon pairs were available for analysis: *Octadecabacter antarcticus* 307 and *Rhodobacter sphaeroides* ATCC 17025, *Glaciecola psychrophila* 170 and *Glaciecola agarylitica* 4H37YE5, *Octadecabacter arcticus* 238 and *Ketogulonicigenium vulgare* Y25, and *Terriglobus roseus* DSM18391 and *Terriglobus saanensis* SP1PR4. Because multiple genes were present in some of these genomes, a total of nine pairwise comparisons were possible. In all nine comparisons the psychrophile protein had a lower isoelectric point and arginine content than the mesophile protein, while valine was elevated in psychrophiles in all comparisons; asparagine and threonine were elevated in psychrophile proteins in all but one comparison (Table 3).

**Table 3:** **Pairwise parameters for candidate alkane hydroxylases in two conserved pfam domains, p450 and Pry_redox_3**

pfam domain	Protein region	Protein parameter	Total number of comparisons^1^	Cases where psychrophile was higher
p450	Whole	Isoelectric point	9	0
p450	Whole	Asparagine	9	8
p450	Whole	Arginine	9	0
p450	Whole	Threonine	9	8
p450	Whole	Valine	9	9
p450	Coil	Isoleucine	9	8
p450	Coil	Aspargine	9	9
p450	Coil	Valine	9	9
p450	β-sheet	Flexibility	9	8
p450	β-sheet	Isoelectric point	9	8
p450	β-sheet	Alanine	9	8
p450	β-sheet	Glycine	9	9
p450	β-sheet	Isoleucine	9	1
p450	β-sheet	Proline	9	9
p450	α-helix	Flexibility	9	8
p450	α-helix	Aspartic acid	9	9
p450	α-helix	Isoleucine	9	0
p450	α-helix	Arginine	9	0
Pyr_redox_3	Whole	Cysteine	11	10
Pyr_redox_3	Whole	Glutamic acid	11	0
Pyr_redox_3	Whole	Valine	11	10
Pyr_redox_3	Coil	Aspartic acid	11	10
Pyr_redox_3	Coil	Glycine	11	1
Pyr_redox_3	β-sheet	GRAVY	11	10
Pyr_redox_3	β-sheet	Cysteine	11	11
Pyr_redox_3	α-helix	Alanine	11	10
Pyr_redox_3	α-helix	Glutamic acid	11	0
Pyr_redox_3	α-helix	Asparagine	11	1

Amino acids in the parameter column refer to amino acid content.

^1^All psychrophile candidate alkane hydroxylases within a pfam were compared with all mesophile candidate alkane hydroxylases in that pfam for a given protein secondary structure (region), protein parameter, and taxonomic pair, where the total number of comparisons possible was 9 or 11 (see Table 2).

There were only two psychrophile-mesophile pairs available for the analysis of putative alkane hydroxylases from the Pyr_redox_3 family: *G. psychrophila* 170 and *G. agarylitica* 4H37YE5 and *Psychrobacter cryohalolentis* K5 and *Acinetobacter baumonii* AYE. Due to the large number of putative hydroxylases belonging to this pfam in *G. psychrophila* 170 and *A. baumonii* AYE, however, 11 comparisons were possible (Table 3). Cysteine and valine were elevated in the psychrophile proteins for all but one comparison; glutamic acid was reduced in the psychrophile proteins for all comparisons. For FA_desaturase only one taxon pair was available for analysis: *O. antarcticus* 307 and *R. sphaeroides* ATCC 17025, with four possible comparisons. Given the limited number of comparisons pairwise FA_desaturase parameters were not explored further.

Considering protein parameters by the secondary structure elements α-helix, β-sheet, or coil also revealed no statistically significant differences in protein physical parameters. The strongest trends were observed for psychrophile FA_desaturases: lowered flexibility in the coil and α-helix regions and reduced acidic residues and lysine in the α-helices. Considering taxon pairs for p450 (Table 3), isoleucine was generally reduced in psychrophile α-helices and β-sheets but elevated in coils. Asparagine and valine were always higher in the coil region for psychrophiles. Flexibility, isoelectric point, alanine, glycine, and proline were all generally elevated in β-sheets. Flexibility and aspartic acid were elevated in α-helices, while arginine was reduced. For psychrophile Pyr_redox_3 (Table 3), aspartic acid was elevated in the coil while glycine was reduced. In psychrophile β-sheets, GRAVY and cysteine were both elevated. Glutamic acid and asparagine were reduced in psychrophile α-helices while alanine was elevated.

**Figure 3:**
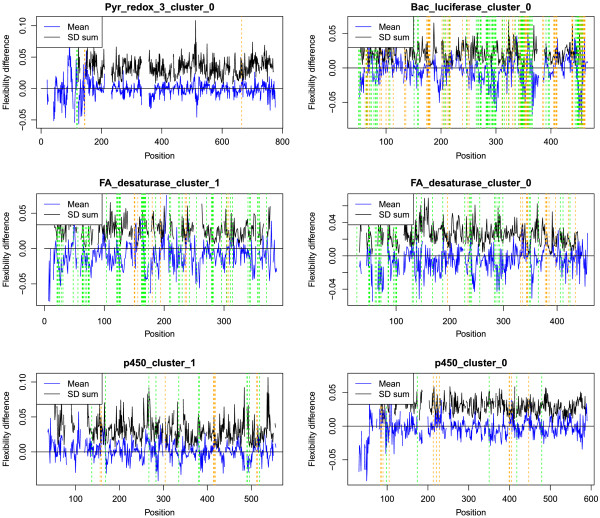
**Alignment of the flexibility parameter between putative alkane hydroxylases in psychrophiles and mesophiles.** Blue line indicates the difference in mean flexibility for the psychrophile and mesophile proteins, black line indicates the sum of the standard deviations for these two groups. Positive values for the mean (blue line) indicate positions in the alignment where the flexibility was greater for the psychrophile proteins; negative values, where flexibility was reduced. Gaps in the data reflect gaps in the alignment that prevented the calculation of the mean or standard deviation (SD). Center residues for windows with a significant increase in flexibility for psychrophiles and mesophiles are indicated by orange and green vertical dashed lines, respectively.

Local differences in flexibility between psychrophile and mesophile proteins within each cluster were apparent after alignment of all the proteins within each cluster (Figure 3), although there was considerable variation in the number of significant differences between clusters and in the direction of the differences (whether greater or lesser flexibility in the psychrophile data set). The Pyr_redox_3 cluster 0 had only five sites with significant differences (see vertical orange and green lines in Figure 3), while the other clusters, Bac_luciferase cluster 0, FA_desaturase cluster 1, FA_desaturase cluster 0, p450 cluster 1, and p450 cluster 1, had many more such sites: 127, 65, 52, 20 and 16, respectively. Summing the difference in mean values (blue line, Figure 3) returned a positive value for p450 cluster 1 (higher degree of flexibility in the psychrophile proteins analyzed) and a negative value for the other clusters (higher degree of flexibility in the mesophile proteins). Restricting this analysis to only those sites with a significant difference in means (sites with an orange or green line, Figure 3) produced a similar result: p450 cluster 0 and p450 cluster 1 yielded a positive sum while the remaining clusters gave a negative sum.

**Figure 4:**
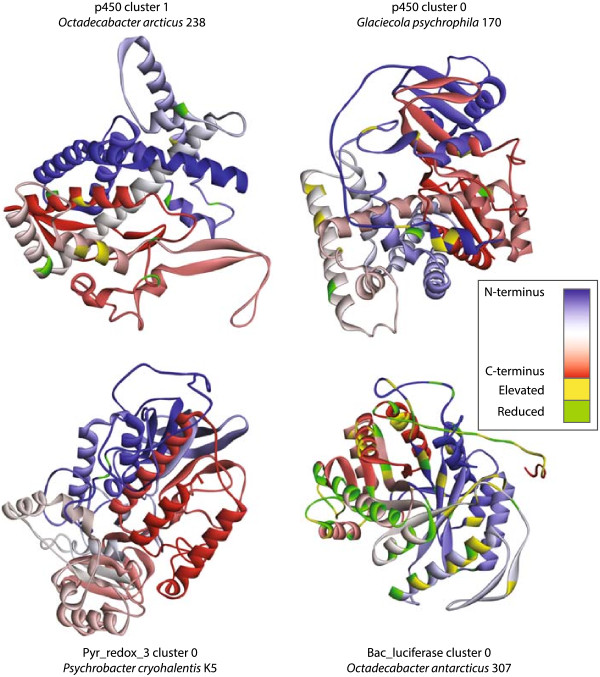
**Predicted 3D structures for representatives of the four clusters from psychrophiles with high confidence predictions.** Proteins are colored from C-terminal (red) to N-terminal (blue). Positions indicated by the orange and green vertical lines in Figure 3 are highlighted as yellow (increased flexibility in psychrophiles) or green (reduced flexibility in psychrophiles).

The Phyre2 protein fold prediction server produced high confidence (90% or more residues modeled at 90% or greater confidence) for the Pyr_redox_3, Bac_luciferase, and p450 pfams. FA_desaturase could not be modeled with high confidence. By correlating the residues in modeled proteins (Figure 4) with positions with a significant difference in mean flexibility, we identified sites that may reflect mutations that enhance protein activity at low temperature (Figure 4). PDB files of the modeled proteins are provided as Additional file 3.

## Discussion

Alkanes are ubiquitous in marine and soil environments, occurring as by-products of cell metabolism and death; they also enter the marine environment from natural hydrocarbon seeps and anthropogenic sources. As a result alkane degradation is likely widespread among heterotrophic bacteria [14]. Several studies have demonstrated that some psychrotolerant Bacteria (growth at 0°C but maximum growth temperature > 20°C), which overlap ecologically and geographically with psychrophilic bacteria, can catabolize alkanes [12, 13, 40, 41], while crude oil degradation has been observed in a variety of cold environments [2, 5, 6, 8–10]. Despite the ubiquity of low molecular weight alkanes, the AMO, AmoC, MeMO_Hyd_G, Monooxygenase_B, and Phenol_Hydrox pfams were not detected in the psychrophile genomes examined. Because these pfams are known to be restricted to relatively few taxonomic groups, their absence in the analyzed psychrophiles, though indicative of an inability to catabolize low molecular weight alkanes in this group, may not be surprising. The nineteen psychrophile genomes available to explore in this analysis, however, represent only a very small sampling of psychrophile functional diversity. Psychrophiles are known, for example, to undergo C1 metabolism [41], yet none of these strains has been targeted for genome sequencing. As more psychrophile genomes are sequenced and published, they can be explored for additional alkane catabolism pathways predicted from environmental evidence but missing in the current set of analyzed genomes. Given that the diversity of enzymes involved in alkane degradation is also not fully explored, the current genomes may well contain alkane hydroxylases lacking sufficient sequence homology to known alkane hydroxylases. This possibility was highlighted by a recent genomic study of a cold-adapted *Colwellia* strain obtained from the deep hydrocarbon plume in the Gulf of Mexico [42]. No genes for known alkane hydroxylase were identified in this strain despite abundant ancillary data linking *Colwellia* to short chain alkane degradation following the Macondo Well blowout [42].

Because alkane bioavailability is positively correlated with temperature and negatively correlated with chain length, the preferential degradation of short chain alkanes is expected in cold environments. Surprisingly, we found several candidate alkane hydroxylases homologous to LadA and AlmA, enzymes associated with the degradation of long-chain alkanes. These putative long-chain alkane hydroxylases are ecologically diverse, occurring in the genomes of sea ice Bacteria (*Octadecabacter arcticus, O. antarcticus*, and *Glaciecola psychrophila*) and tundra soil Bacteria (*Terroglobus saanensis*). Confirming the ability of these strains to degrade long-chain alkanes or similar substrates will be a priority in future work. Because *O. arcticus*, *O. antarcticus*, and *G. psychrophila* are all associated with sea ice (Table 2), which in springtime hosts a high density of ice algae, their hypothesized ability to degrade long-chain alkanes may result from a preference for ice-algal lipids. The bioavailability of lipids and long-chain alkanes can be enhanced at low temperature by naturally occurring surfactants, most notably microbially produced exopolymers (EPS) [43]. Sea ice is rich in EPS [44] which may enable the catabolism of these compounds even at low temperature.

A considerable body of literature is dedicated to determining what protein modifications enable enzymatic function at low temperature. At low temperatures water molecules interact more tightly with the protein surface, reducing the overall flexibility of the protein. To counter the effect of low temperature on enzyme function, cold-active proteins make use of a variety of amino acid substitutions. The sum impact of these different substitutions, including their interactions and feedbacks, is difficult to predict. Compounding this difficulty is the co-occurrence of low temperature and low water activity, as found in virtually all ice matrices (e.g., permafrost, glacial ice, and sea ice). Optimization to low water activity and low temperature may be more difficult than optimization to low temperature alone.

Although all analyzed pfams showed some differences between psychrophiles and mesophiles for the measured parameters, no coherent overall optimization strategy was evident. The clearest trends appeared in our pairwise comparisons, which were limited to Pyr_redox_3 and p450. High flexibility and low isoelectric point appeared to be important for cold adaptation in p450, where asparagine, threonine, and valine were all enriched and arginine reduced. For Pyr_redox_3, cysteine and valine were enriched and glutamic acid was reduced. These modifications differ somewhat from those described in previous work on amino acid substitutions among cold-adapted proteins. Working with a more limited number of psychrophile genomes, and without distinguishing between protein families, Metpally and Reddy [45] did not report a role for valine or cysteine in protein cold adaptation. Our findings suggest that amino acid substitution patterns may require a more nuanced view, differing between proteins as a result of protein structure, strain taxonomy, and ecology.

One challenge to evaluating protein temperature optimization is the localization of some parameters. Although changes to isoelectric point and hydropathy are likely to be globally distributed in a protein, at least among secondary structure elements or among sites of a given solvent accessibility, optimized flexibility may come about through the modification of only specific residues [46, 47]. Regions of consistently increased flexibility were present in alignments from all six putative alkane hydroxylase clusters, though the generalized location of increased flexibility varied between cluster representatives (Figure 4). P450 cluster 0 had several regions of increased flexibility at probable hinge points on bends, loops, and in the coil region. Interestingly, three of these were centered on methionine residues (Met8, Met269, and Met295 in the representative p450 cluster 0 protein from *Glaciecola psychrophila*). Methionine is known to play a role in low temperature optimization of other heme-binding proteins by providing alternate heme-binding sites in the event of partial denaturation [47]. In the *G. psychrophila* p450, however, most of these sites were located toward the exterior of the protein and are unlikely to interact with heme. P450 cluster 1 had no evidence of increased flexibility in loops or coils, but did have regions of increased flexibility in the core of the protein. Bac_luciferase cluster 0 had large differences in local flexibility between the psychrophile and mesophile proteins. Regions of increased flexibility included bends likely to function as hinge points and residues near the protein active site.

## Conclusions

Although the total number of putative alkane hydroxylases in the analyzed psychrophiles was smaller than in a taxonomically related group of mesophiles, the metabolic potential for alkane degradation in the psychrophiles is clear. These findings are consistent with environmental observations of crude oil degradation in sea ice, permafrost, and most recently the cold deep ocean. As in other cold-active enzymes, the putative alkane hydroxylases show clear and, within clusters, consistent differences in amino acid composition and protein parameters from mesophilic homologues. These proteins are good candidates for rate studies, such as enzyme assays using fluorescently labeled substrates, and rational manipulations, such as targeted mutation for enhancement of the substrate range or optimal physicochemical conditions.

## Electronic supplementary material

Additional file 1: Figure S1: Maximum-likelihood tree of 16S rRNA genes for strains used in this analysis. (PDF 170 KB)

Additional file 2: Table S1: Genbank accession and annotation for the protein records corresponding to the gene products evaluated in this study. (TXT 10 KB)

Additional file 3: **pdb_files.** PDB files of the predicted structures for the four proteins in Figure 4. (ZIP 235 KB)

## References

[1] Hazen TC, Dubinsky EA, DeSantis TZ, Andersen GL, Piceno YM, Singh N, Jansson JK, Probst A, Borglin SE, Fortney JL, Stringfellow WT, Bill M, Conrad ME, Tom LM, Chavarria KL, Alusi TR, Lamendella R, Joyner DC, Spier C, Baelum J, Auer M, Zemla ML, Chakraborty R, Sonnenthal EL, D'Haeseleer P, Holman H-YN, Osman S, Lu Z, Van Nostrand JD, Deng Y (2010). Deep-sea oil plume enriches indigenous oil-degrading bacteria. Science.

[2] Redmond MC, Valentine DL (2012). Natural gas and temperature structured a microbial community response to the Deepwater Horizon oil spill. P Natl Acad Sci.

[3] Valentine DL, Kessler JD, Redmond MC, Mendes SD, Heintz MB, Farwell C, Hu L, Kinnaman FS, Yvon-Lewis S, Du M, Chan EW, Tigreros FG, Villanueva CJ (2010). Propane respiration jump-starts microbial response to a deep oil spill. Science.

[4] Colwell RR, Walker JD, Cooney JJ (1977). Ecological aspects of microbial degradation of petroleum in the marine environment. CRC Cr Rev Microbiol.

[5] Brakstad O, Nonstad I, Faksness L-G, Brandvik P (2008). Responses of microbial communities in Arctic Sea Ice after contamination by crude petroleum oil. Microb Ecol.

[6] Gerdes B, Brinkmeyer R, Dieckmann G, Helmke E (2005). Influence of crude oil on changes of bacterial communities in Arctic sea-ice. FEMS Microb Ecol.

[7] Delille D, Basseres A, Dessommes A (1996). Seasonal variation of bacterial in sea ice contaminated by diesel fuel and dispersed crude oil. Microb Ecol.

[8] Bell TH, Yergeau E, Maynard C, Juck D, Whyte LG, Greer CW (2013). Predictable bacterial composition and hydrocarbon degradation in Arctic soils following diesel and nutrient disturbance. ISME J.

[9] Margesin R, Labbe D, Schinner F, Greer C, Whyte L (2003). Characterization of hydrocarbon-degrading microbial populations in contaminated and pristine alpine soils. Appl Environ Microbiol.

[10] Delille D (2000). Response of Antarctic soil bacterial assemblages to contamination by diesel fuel and crude oil. Microb Ecol.

[11] Powell S, Bowman J, Snape I (2004). Degradation of nonane by bacteria from Antarctic marine sediment. Pol Biol.

[12] Whyte LG, Bourbonniere L, Greer CW (1997). Biodegradation of petroleum hydrocarbons by psychrotrophic Pseudomonas strains possessing both alkane (alk) and naphthalene (nah) catabolic pathways. Appl Environ Microbiol.

[13] Whyte LG, Hawari J, Zhou E, Bourbonnière L, Inniss WE, Greer CW (1998). Biodegradation of variable-vhain-length alkanes at low temperatures by a psychrotrophic *Rhodococcus* sp. Appl Environ Microbiol.

[14] Nie Y, Chi C-Q, Fang H, Liang J-L, Lu S-L, Lai G-L, Tang Y-Q, Wu X-L (2014). Diverse alkane hydroxylase genes in microorganisms and environments. Sci Reports.

[15] Glieder A, Farinas ET, Arnold FH (2002). Laboratory evolution of a soluble, self-sufficient, highly active alkane hydroxylase. Nat Biotech.

[16] Harford-Cross CF, Carmichael AB, Allan FK, England PA, Rouch DA, Wong L-L (2000). Protein engineering of cytochrome P450cam (CYP101) for the oxidation of polycyclic aromatic hydrocarbons. Protein Eng.

[17] Wentzel A, Ellingsen T, Kotlar H-K, Zotchev S, Throne-Holst M (2007). Bacterial metabolism of long-chain n-alkanes. Appl Microbiol Biotechnol.

[18] Beilen JB, Funhoff EG (2007). Alkane hydroxylases involved in microbial alkane degradation. Appl Microbiol Biotechnol.

[19] Van Beilen JB, Li Z, Duetz WA, Smits TH, Witholt B (2003). Diversity of alkane hydroxylase systems in the environment. Oil Gas Sci Technol.

[20] Ji Y, Mao G, Wang Y, Bartlam M (2013). Structural insights into diversity and n-alkane biodegradation mechanisms of alkane hydroxylases. Front Microbiol.

[21] Throne-Holst M, Wentzel A, Ellingsen TE, Kotlar H-K, Zotchev SB (2007). Identification of novel genes involved in long-chain n-alkane degradation by Acinetobacter sp. strain DSM 17874. Appl Environ Microbiol.

[22] Feng L, Wang W, Cheng J, Ren Y, Zhao G, Gao C, Tang Y, Liu X, Han W, Peng X (2007). Genome and proteome of long-chain alkane degrading Geobacillus thermodenitrificans NG80-2 isolated from a deep-subsurface oil reservoir. Proc Natl Acad Sci.

[23] Bairoch A, Bougueleret L, Altairac S, Amendolia V, Auchincloss A, Argoud-Puy G, Axelsen K, Baratin D, Blatter M, Boeckmann B (2009). The Universal Protein Resource (UniProt) 2009. Nuc Acids Res.

[24] Eddy SR (1998). Profile hidden Markov models. Bioinformatics.

[25] Punta M, Coggill PC, Eberhardt RY, Mistry J, Tate J, Boursnell C, Pang N, Forslund K, Ceric G, Clements J, Heger A, Holm L, Sonnhammer ELL, Eddy SR, Bateman A, Finn RD (2012). The Pfam protein families database. Nuc Acids Res.

[26] Bernal A, Ear U, Kyrpidesa N (2001). Genomes OnLine Database (GOLD): a monitor of genome projects world-wide. Nuc Acids Res.

[27] **hima: A Meta-database of Low Temperature Genomes and Metagenomes** [http://reric.org/work/hima/]

[28] Casanueva A, Tuffin M, Cary C, Cowan DA (2010). Molecular adaptations to psychrophily: the impact of ‘omic’ technologies. Trends Microbiol.

[29] Siddiqui KS, Williams TJ, Wilkins D, Yau S, Allen M, Brown MV, Lauro FM, Cavicchioli R (2013). Psychrophiles. Ann Rev Earth Pl Sc.

[30] Sievers F, Wilm A, Dineen D, Gibson TJ, Karplus K, Li W, Lopez R, McWilliam H, Remmert M, Söding J (2011). Fast, scalable generation of high-quality protein multiple sequence alignments using Clustal Omega. Mol Syst Biol.

[31] Kimura M (1980). A simple method for estimating evolutionary rates of base substitutions through comparative studies of nucleotide sequences. J Mol Evol.

[32] Oksanen J, Blanchet FG, Kindt R, Legendre P, Minchin PR, O’Hara RB, Simpson GL, Solymos P, Henry M, Stevens H, Wagner H (2013). vegan: Community Ecology Package. R package version 2.0-10.

[33] Pelé J, Abdi H, Moreau M, Thybert D, Chabbert M (2011). Multidimensional scaling reveals the main evolutionary pathways of class A G-protein-coupled receptors. PLoS ONE.

[34] Price M, Dehal P, Arkin A (2010). FastTree 2 - Approximate maximum likelihood trees for large alignments. PLoS ONE.

[35] Cock PJ, Antao T, Chang JT, Chapman BA, Cox CJ, Dalke A, Friedberg I, Hamelryck T, Kauff F, Wilczynski B (2009). Biopython: freely available Python tools for computational molecular biology and bioinformatics. Bioinformatics.

[36] Ikai A (1980). Thermostability and aliphatic index of globular proteins. J Biochem.

[37] McGuffin LJ, Bryson K, Jones DT (2000). The PSIPRED protein structure prediction server. Bioinformatics.

[38] Kelley LA, Sternberg MJ (2009). Protein structure prediction on the Web: a case study using the Phyre server. Nat Protocols.

[39] Holm S (1979). A simple sequentially rejective multiple test procedure. Scand J Stat.

[40] Cao B, Ma T, Ren Y, Ren Y, Li G, Li P, Guo X, Ding P, Feng L (2011). Complete genome sequence of Pusillimonas sp. T7-7, a cold-tolerant diesel oil-degrading bacterium isolated from the Bohai Sea in China. J Bacteriol.

[41] Trotsenko YA, Khmelenina VN (2002). Biology of extremophilic and extremotolerant methanotrophs. Arch Microbiol.

[42] Mason O, Han J, Woyke T, Jansson J (2014). Single-cell genomics reveals features of a *Colwellia* species that was dominant during the Deepwater Horizon oil spill. Front Microbiol.

[43] Gutierrez T, Berry D, Yang T, Mishamandani S, McKay L, Teske A, Aitken MD (2013). Role of bacterial exopolysaccharides (EPS) in the fate of the oil released during the deepwater horizon oil spill. PLoS ONE.

[44] Krembs C, Eicken H, Junge K, Deming JW (2002). High concentrations of exopolymeric substances in Arctic winter sea ice: implications for the polar ocean carbon cycle and cryoprotection of diatoms. Deep Sea Res Part I.

[45] Metpally R, Reddy B (2009). Comparative proteome analysis of psychrophilic versus mesophilic bacterial species: Insights into the molecular basis of cold adaptation of proteins. BMC Genomics.

[46] Leiros H-KS, Pey AL, Innselset M, Moe E, Leiros I, Steen IH, Martinez A (2007). Structure of phenylalanine hydroxylase from *Colwellia psychrerythraea* 34H, a monomeric cold active enzyme with local flexibility around the active site and high overall stability. J Biol Chem.

[47] Harvilla PB, Wolcott HN, Magyar JS (2014). The structure of ferricytochrome c 552 from the psychrophilic marine bacterium *Colwellia psychrerythraea* 34H. Metallomics.

