# Supplementary figures and images for: Alkane hydroxylase genes in psychrophile genomes and the potential for cold active catalysis
Source: BMC Genomics. 2014 Dec 16;15(1):1120. doi: 10.1186/1471-2164-15-1120 (PMC4320567; doi:10.1186/1471-2164-15-1120)

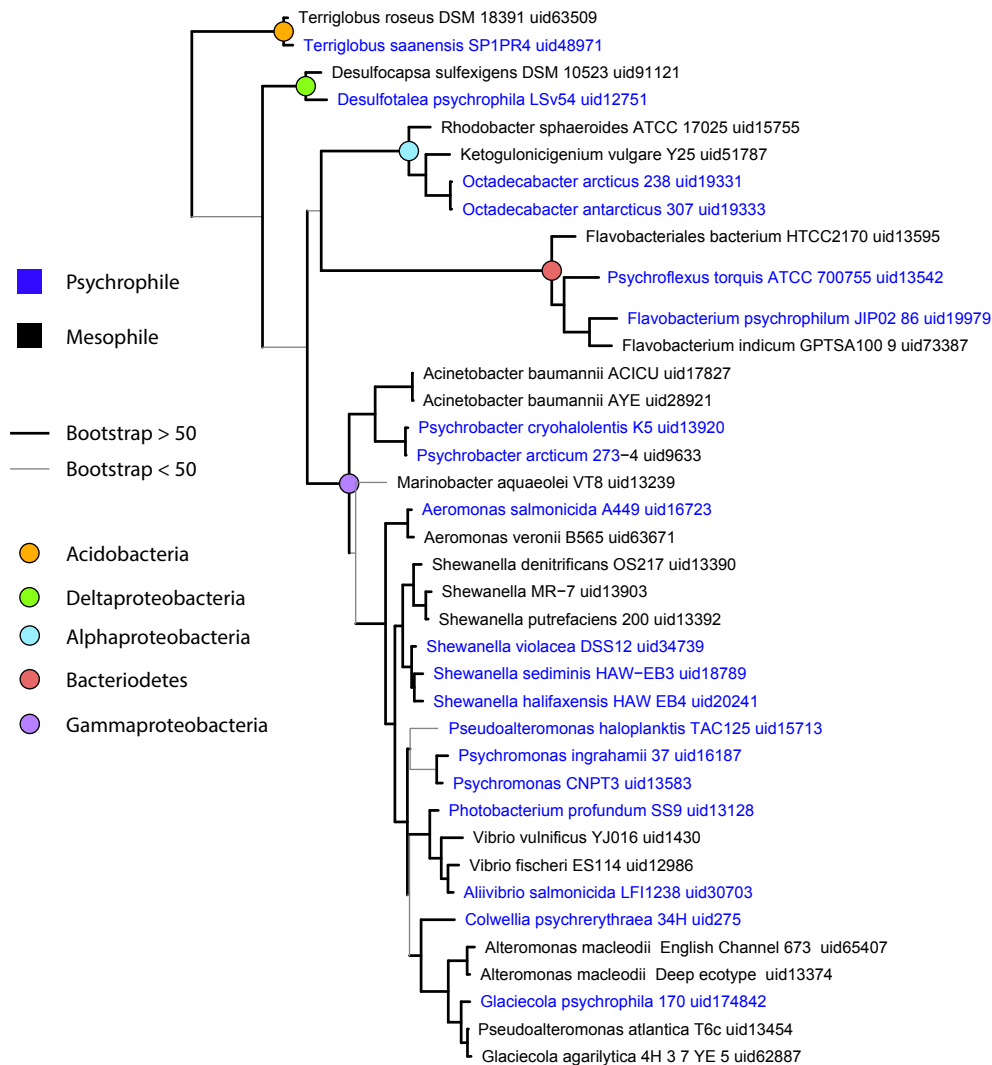

Supplement: Supplementary file 1 — Additional file 1: Figure S1: Maximum-likelihood tree of 16S rRNA genes for strains used in this analysis. (PDF 170 KB) [file 12864_2014_6905_MOESM1_ESM.pdf]
